# Verteporfin-induced lysosomal compartment dysregulation potentiates the effect of sorafenib in hepatocellular carcinoma

**DOI:** 10.1038/s41419-019-1989-z

**Published:** 2019-10-03

**Authors:** Jacopo Gavini, Noëlle Dommann, Manuel O. Jakob, Adrian Keogh, Laure C. Bouchez, Sofia Karkampouna, Marianna Kruithof-de Julio, Michaela Medova, Yitzhak Zimmer, Anna M. Schläfli, Mario P. Tschan, Daniel Candinas, Deborah Stroka, Vanessa Banz

**Affiliations:** 1 Department of Visceral Surgery and Medicine, Department for BioMedical Research, Inselspital, Bern University Hospital and University of Bern, 3010 Bern, Switzerland; 2 Graduate School for Cellular and Biomedical Sciences, University of Bern, Bern, Switzerland; 3 Glenmark Pharmaceuticals, 1003 Lausanne, Switzerland; 4 Department of Urology, Department for BioMedical Research, Inselspital, Bern University Hospital and University of Bern, 3010 Bern, Switzerland; 5 Department of Radiation Oncology, Department for BioMedical Research, Inselspital, Bern University Hospital and University of Bern, 3010 Bern, Switzerland; 6 Institute of Pathology, University of Bern, Murtenstrasse 31, 3008 Bern, Switzerland

**Keywords:** Liver cancer, Mechanisms of disease

## Abstract

Lysosomal sequestration of anti-cancer compounds reduces drug availability at intracellular target sites, thereby limiting drug-sensitivity and inducing chemoresistance. For hepatocellular carcinoma (HCC), sorafenib (SF) is the first line systemic treatment, as well as a simultaneous activator of autophagy-induced drug resistance. The purpose of this study is to elucidate how combination therapy with the FDA-approved photosensitizer verteporfin (VP) can potentiate the antitumor effect of SF, overcoming its acquired resistance mechanisms. HCC cell lines and patient-derived in vitro and in vivo preclinical models were used to identify the molecular mechanism of action of VP alone and in combination with SF. We demonstrate that SF is lysosomotropic and increases the total number of lysosomes in HCC cells and patient-derived xenograft model. Contrary to the effect on lysosomal stability by SF, VP is not only sequestered in lysosomes, but induces lysosomal pH alkalinization, lysosomal membrane permeabilization (LMP) and tumor-selective proteotoxicity. In combination, VP-induced LMP potentiates the antitumor effect of SF, further decreasing tumor proliferation and progression in HCC cell lines and patient-derived samples in vitro and in vivo. Our data suggest that combination of lysosome-targeting compounds, such as VP, in combination with already approved chemotherapeutic agents could open a new avenue to overcome chemo-insensitivity caused by passive lysosomal sequestration of anti-cancer drugs in the context of HCC.

## Introduction

Autophagy and lysosomes play an important role in all eukaryotic cells, by restoring homeostasis after intra- or extracellular stress conditions^1,2^. They are both part of the intracellular catabolic machinery, often driving chemotherapy resistance, as well as tumor proliferation^1,3,4^. Nadanaciva et al. showed that several compounds and anticancer drugs, including tyrosine kinase inhibitors (TKIs), such as sunitinib, have a high affinity for the lysosomal compartment due to their chemical structure^5^. The process by which chemotherapeutic agents undergo intracellular compartmentalization within the lumen of lysosomes is called lysosomotropism and it can reduce their potency at the specific target sites^6–8^. The acidic environment of the lysosomes, together with the fact that chemotherapeutics are often formulated as weak-bases, enables their accumulation within their lumen by simple diffusion. Immediately after their internalization, lysosomotropic compounds become protonated, resulting in their lysosomal compartmentalization^9–12^. Various strategies are being sought to overcome lysosome-mediated chemoresistance, such as lysosomal photodestruction, targeting lysosomal acid sphingomyelinase (ASM) and inducing lysosomal membrane permeabilization (LMP)^9,13–16^. In hepatocellular carcinoma (HCC), a notoriously difficult to treat tumor^17^ and second leading cause of cancer-related deaths worldwide^18^, autophagy and lysosomes seem to play a pivotal role in chemotherapy resistance against the oral FDA-approved first line TKI sorafenib (SF)^19,20^. Indeed, SF was shown to induce autophagy as an adaptive response mechanism as well as to be less effective on HCC cells with larger or increased numbers of lysosomes at basal level^19,20^. Due to tumor cell adaptive responses, development of SF-induced resistance is one of the major causes of treatment failure in patients with advanced HCC^6,17^. Verteporfin (VP), is a benzoporphyrin derivate belonging to the porphyrin family and already used as a photosensitizer for photodynamic therapy in macular degeneration^21^. For cancer therapy, VP was initially used as a photosensitizer, able to eliminate tumor cells by the production of oxygen radicals by non-thermal light activation^21,22^. VP has also been investigated in the absence of light, showing anti-tumor properties due to the inhibition of autophagosome formation as well as tumor-specific proteotoxicity, independently of its described target Yes-associated protein 1 (YAP1)^23–26^. Here we report how VP-induced lysosomal membrane permeabilization can circumvent intracellular catabolic factors limiting the efficacy of SF. Our findings reveal that by specifically targeting the tumor lysosomal compartment stability and autophagic flux progression, VP can unravel a new strategy to overcome the chemo-insensitivity of SF in the context of HCC.

## Materials and methods

### Cell lines and primary human hepatocyte cultures

All HCC cell lines were purchased from American Type Culture Collection (ATCC - LCG Promochem) and cultured either in Dulbecco’s Modified Eagle’s or RPMI medium 1640 (Life Technology) with 10% of FBS, 100 U/mL Penicillin and 100 μg/mL Streptomycin (Life Technology). Human immortalized microvascular endothelial cells (HMEC-1) were purchased from ATCC and cultured in MCDB 131 medium with 5% of FBS, 10 ng/mL of HEGF (Sigma-Aldrich), 1 ng/mL of hydrocortisone (Sigma-Aldrich) and antibiotics. Murine fibroblast NIH3T3 MET M1268T cell lines stably and ectopically expressing K-RAS (G12D, G12V, G13D) and H-RAS (G12V) mutations were kindly provided by Prof. Yitzhak Zimmer (Department for BioMedical Research (DBMR), University of Bern, Switzerland) and maintained in DMEM (Gibco) supplemented with 10% FBS and antibiotic-antimycotic 0.5 mg/ml Geneticin/G-418 sulfate (Gibco)^27^. Normal liver tissue was obtained from surgical resections from consented patients at the University Hospital of Bern. Hepatocytes were isolated by enzymatic perfusion, as previously described^28^. All cultured cells were kept at 37 °C in a humidified incubator with 5% of CO_2_.

### Chemicals and reagents

Stock solutions of verteporfin (VP - SML0534 - Sigma-Aldrich) were dissolved in dimethyl sulfoxide (DMSO – 10 mmol/L) at the time of use. Resazurin sodium salt (R7017 - Sigma-Aldrich) was prepared in sterile distilled water (15 mmol/L) to be used as an indicator of cell viability in mammalian cell cultures. BAY 54-9085 (sorafenib tosylate - SF) was obtained from Bayer HealthCare Pharmaceuticals, chloroquine (CQ - C6628), bafilomycin A1 (BafA - B1793) and Leu-Leu methyl ester hydrobromide (LLOMe – L7393) were all purchased from Sigma-Aldrich.

### Cell proliferation and cytotoxicity assays

Cells (2,5 × 10^3^ per well) were seeded in 96-well flat bottom plates. All the cells were treated as indicated, taking as a control cells treated with the appropriate vehicle. For Resazurin cell viability assay, the viability of treated cells was measured by adding 10% of the media volume of the Resazurin stock solution for 2 h. Luminescence was measured with Tecan Infinite® 200 Plate Reader at wavelengths 545 nm and 590 nm. The coefficient of drug interaction (CDI) was calculated as previously described^29^. Cytotoxicity was assessed using an LDH cytotoxicity detection kit (Roche) according to the manufacturer’s guidelines. All the experiments were performed in darkness and treated cells were protected from light using aluminum foil in order to avoid VP photo-activation. All experiments were performed in triplicates.

### RNA extraction and quantitative real-time RT-PCR (qPCR)

RNA was isolated from cells as well as tissue samples by TRIzol Reagent following the manufacture’s protocol (Life Tecnologies). cDNA was synthesized using Omniscript RT Kit 200 (Qiagen). mRNA was analyzed by reverse transcriptase-quantitative PCR (RT-qPCR; ABI 7900, SDS 2.4 software). Expression levels of human genes of interest were measured by TaqMan mastermix (Roche). All the primers used were from Thermo Fisher Scientific.

### Cell-cycle analysis by Flow cytometry

Cells fixed with 70% EtOH were incubated for 2 h with RNase A (40 μg – Promega)/Propidium iodide solution (50 μg–Sigma-Aldrich). Cell-cycle distribution was determined by fluorescence-activated cell sorting FACS using the LSR II and FACSDiva software (Becton Dickinson). All experiments were performed with three samples per condition.

### Angiogenesis tube formation assay in 3D Matrigel®

HMEC-1 cells were seeded at a density of 1.5 × 10^4^ cells per well in a 96-well plate, previously coated with 30 μL of Matrigel® (DB Biosciences). Pictures were taken with a Leica camera and afterwards analyzed by a semi-automated plug-in for ImageJ software as previously described by Gilles Carpentier (2012). All experiments were performed in triplicate.

### Live-cells fluorescence microscopy and Lysotracker fluorescence assay

For live-cell imaging, HCC cell lines and primary human hepatocytes were seeded (1.5 × 10^4^ cells per well) in a chambered coverglass (Thermo Scientific) and treated as indicated before. After 24 h cells were washed twice in cold DPBS and stained with LysoTracker DNS-99 (Thermo Fisher). Nuclei were counterstained with DAPI (1:5000). Verteporfin auto-fluorescence was observed in the far-red spectrum (Cy5 channel). Fluorescence images of live-cells were obtained using an automated inverted microscope (Leica DMSI4000 B). For the Lysotracker fluorescence assay, HCC cells were seeded (1.5 × 10^4^ cells per well) on a 96-well assay plate and treated as described. After 24 h, medium was aspirated and discarded from all the wells followed by Lysotracker incubation as previously reported^30^. LysoTracker fluorescence intensity was then measured by spectrophotometry (Ex = 577 nm, Em = 590 nm) on a fluorescence plate reader (Tecan Infinite® 200).

### Calcium phosphate transfection and GFP-WIPI-1 puncta analysis

For transient calcium phosphate transfection, HuH7 and HepG2 cells were seeded on coverslips and grown to 70–80% confluence in culture medium at 37 °C and 5% CO_2_. Cells were transfected as previously shown^31^ with GFP-WIPI-1 plasmid DNA (kindly provided by Prof. Tassula Proikas-Cezanne, University of Tübingen). Approximately 24 h later, transfected cells were treated as described, washed with PBS and fixed in 4% formaldehyde. Nuclei were counterstained with DAPI (1:5000) and analyzed by confocal microscopy (Olympus FV1000, Olympus, Volketswil, Switzerland) at 60x magnification. The number of GFP-WIPI-1 dots was quantified using ImageJ software and using a modified version of the plugin described elsewhere^32^.

### Immunocytochemistry

HCC cell lines were grown on glass cover slips in 24-well plates (2 × 10^4^ cells per well) and treated as indicated before. Cells were fixed with 4% formaldehyde, permeabilized and blocked in 5% goat serum (DAKO, X0907), 0.3% Triton-X-100 (Sigma-Aldrich) in DPBS and stained with anti-galectin-1 antibody (Abcam – 1:1000) and matched with Alexa Fluor® (AF) 488 conjugated secondary antibodies (Life Technologies -1:1000). Nuclei were counterstained with DAPI (1:5000), coverslips were mounted with VECTASHIELD Antifade Mounting Medium (Vector Laboratories, H-1000) and fluorescence images were taken using an automated inverted microscope (Leica DMSI4000 B).

### Western blot analysis

All total protein lysates were obtained using RIPA buffer 10 mM Tris, pH8, 1 mM EDTA, pH8, 150 mM NaCl; phosphatase inhibitors (Na3VO4, NaF, PMSF); a protease inhibitor mix (Sigma-Aldrich) and 0.5% NP40. Western blots were performed according to standard protocols. Primary antibodies were purchased as follows: polyclonal anti-LC3B (Novus Biologicals - 1:1000), polyclonal anti-p62/SQSTM1 (Sigma-Aldrich - 1:1000), polyclonal anti-PARP (Cell Signaling – 1:1000), polyclonal anti-caspase 3 (Cell Signaling – 1:1000), monoclonal anti-LAMP-1 (Santa Cruz Biotechnology – 1:1000), monoclonal anti-Hsp70 (Abcam – 1:2000), polyclonal anti-pERK1/2 (Cell Signaling – 1:1000), polyclonal anti-ERK1/2 (Abcam – 1:1000), polyclonal anti-pan RAS (Cell Signaling –1:1000), phosphor-mTOR (Ser 2448; Cell Signaling – 1:1000), mTOR (Cell Signaling – 1:1000); phosphor-S6 kinase (Cell Signaling – 1:1000); S6 kinase (Cell Signaling – 1:1000), phosphor-4E-BP1 (Cell Signaling – 1:1000), 4E-BP1 (Cell Signaling – 1:1000) and monoclonal anti-β-actin-peroxidase clone AC-15 (Sigma Aldrich - 1:100000). HRP-conjugated secondary antibodies were purchased as follows: goat anti-rabbit (1:1000 - Dako), goat anti-mouse (1:1000 - Dako). Membranes were incubated with enhanced chemiluminescence (Western Lightning Plus-ECL - Perkin Elmer) and films were developed by CURIX 60 (AGFA) or Fusion-FX7 SPECTRA (VILBER). The band size was estimated using Precision Plus Protein^TM^ Dual Color Standards (BIO-RAD).

### Lysosomal pH measurements by flow cytometry

Lysosomal pH measurements were based on a detailed protocol recently published by Eriksson et al.^33^. In brief, HCC cell lines and primary human hepatocytes were seeded and cultured in cell culture medium containing FITC-Dextran (0.1 mg/mL - FD40S - Sigma-Aldrich) for three days. After this period FITC-Dextran was removed by aspirating the medium and fresh cell culture medium was added with the respective treatments as indicated. Lysosomal pH from samples belonging to the standard curve scale (pH ranging from 4 to 6) and experimental-treated ones were analyzed by FACS. Triplicate samples were taken for each condition analyzed.

### HCC patient-derived tumoroid cultures

HCC surgical resections were obtained from consented patients at University Hospital of Bern. Enzymatic digestion of minced tissue and 3D tumoroids culture were performed as previously described^34^. For the drug-sensitivity assay, tumoroids were plated in Costar® Ultra-Low Attachment 96-well plate (Corning) with expansion medium supplemented with VP, SF and the respective vehicles. Tumoroid-viability assay was conducted as previously described^34^. Briefly, HCC patient-derived tumoroids (PDT) viability was assessed by CellTiter-Glo® 3D Cell Viability Assay (Promega) after 2 days of drugs incubation, according to the manufacturer’s guidelines. To assess the tumoroid 3D structure and morphology, bright-field images were taken using an automated inverted microscope (Leica DMSI4000 B).

### Subcutaneous HCC cell line and patient-derived xenograft mouse models

Eight- to twelve-week-old male Rag2^−/−^γ_c_^−/−^ mice were used for the subcutaneous xenograft model. In total 2 × 10^6^ HepG2 cells were injected in a 1:1 ratio with Matrigel® (BD Biosciences). HCC patient-derived specimens were transplanted subcutaneously. Tumor volume was measured using a digital caliper thanks to a modified ellipsoid formula: volume = (4/3) × π × (length/2) × (width/2) × (height/2). Upon reaching a volume of 250 mm^3^, animals were randomly divided into different groups as described: verteporfin (100 mg/kg) was administered every second day by intraperitoneal injection; sorafenib (60 mg/kg) daily by oral gavage; combined treatment with VP and SF; administration of the respective vehicles only (control group). The tumor volume and the body weight were recorded every second day for fourteen days. At the end of experiment, the entire tumor was carefully removed and some snap-frozen samples were stored in liquid nitrogen and kept at −80 °C until further use. Other parts of the tissue were fixed in 4% formaldehyde and in Tissue-Tek Optimal Cutting Temperature Compound from Sakura-Finetek® (O.C.T.^TM^) for histological analysis. All animal experiments were conducted in accordance to Swiss Guidelines of Care and Use of Laboratory Animals.

### Immunohistochemistry and immunofluorescence

Tissue samples were fixed in 4% formaldehyde, processed, embedded in paraffin and sectioned with Leica Microtome. Sections were stained using Ki67 antibody (Abcam) and CD31 antibody (Abcam). For each sample three randomly chosen fields were imaged by Leica camera and analyzed by ImageJ software. Cryo-sections (5 μm thick) were incubated with LAMP-1 primary antibody (Santa Cruz Biotechnology) followed by the Anti-Mouse IgG1 FITC (eBioscience) secondary antibody. Verteporfin auto-fluorescence was observed in the far-red spectrum. Coverslips were mounted with VECTASHIELD Antifade Mounting Medium with DAPI (Vector Laboratories, H-1500) and fluorescence images were taken using an automated inverted microscope (Leica DMSI4000 B).

### Statistical analysis

Values are presented as means ± SEM. Statistical differences for single comparisons were determined using unpaired, two-tailed Student t tests, as well as 1-way or 2-way analysis of variance (ANOVA). Tukey’s post-test was performed to examine differences between drug treatment effects. Longitudinal growth in vivo data were used to test the difference of the tumor growth among treatment groups. *P* values < 0.05 were considered statistically significant. Statistical analyses were carried out using GraphPad Prism 7.

## Results

### Verteporfin decreases hepatocellular carcinoma cell viability and potentiates the antitumor effect of sorafenib

**Fig. 1:**
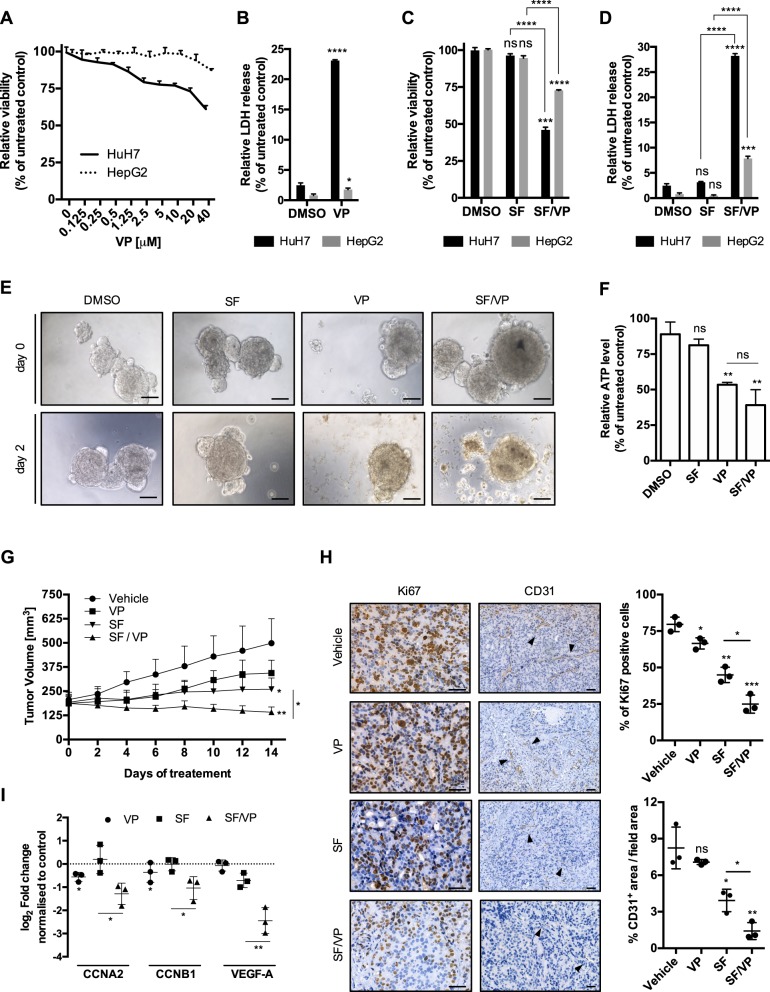
VP potentiates the anti-tumor effect of SF in vitro and in vivo. **a** Mean ± SD of HuH7 and HepG2 relative viability was analyzed after 24 h of VP treatment at different concentrations. Values were normalized to untreated cells, *n* = 3. **b** Mean ± SD relative of HuH7 and HepG2 LDH release after 24 h treatment with verteporfin (VP – 20 μM). Values were normalized to untreated cells. *n* = 3. **c** Mean ± SD of HuH7 and HepG2 relative viability was analyzed after 24 h treatment with sorafenib (SF – 5 μM) or in combination with verteporfin (SF – 5 μM/VP - 20 μM). Values were normalized to untreated cells. *n* = 3. **d** Mean ± SD relative of HuH7 and HepG2 LDH release after 24 h treatment with sorafenib (SF - 5 μM) or in combination with verteporfin (SF – 5 μM/VP – 20 μM). Values were normalized to untreated cells. *n* = 3. **e** Representative bright-field images of HCC patient–derived tumoroids treated with SF (5 μM), VP (20 μM), both drugs combined together (SF/VP) and vehicle-treated for 2 days. Scale bar 50 μm. **f** Mean ± SD of ATP level detected by CellTiter-Glo^®^ 3D Cell Viability Assay (Promega) of HCC patient-derived tumoroids (PDT) after SF (5 μM), VP (20 μM) and both drugs combined together (SF/VP) treatment for 2 days. Values were normalized to vehicle-treated PDT after 2 days, *n* = 3. **g** Tumor volume of HCC patient-derived xenografts mice (*n* = 3/group) treated with vehicle, verteporfin (VP – 100 g/kg – i.p. every second day), sorafenib (SF – 60 g/kg – daily oral gavage) and VP/SF combination for 14 days. **h** HCC PDX immunohistochemistry for Ki67 and CD31 (black arrows) in vehicle, VP, SF and VP/SF treated animals after 14 days. Counterstain: hematoxylin. Scale bar 200 μm. The graphs show the mean percentage ± SD of Ki67 positive cells and CD31 positive areas per tumor/mouse (three random fields were used for the analysis per tumor/mouse). **i** HCC PDX real-time qPCR data of cell-cycle progression (CCNA2 and CCNB1) and vascular endothelial growth factor A (VEGF-A) genes after VP, SF and VP/SF treated animals after 14 days. Values were normalized to the vehicle-treated mice. *P* values < 0.05 were considered statistically significant and are indicated as follows: *P < 0.05; **P < 0.01; ***P < 0.001; ****P < 0.0001; ns, not significant

Here, we tested the effect of VP in HCC, in the absence of its light activation and demonstrated that in HepG2 and HuH7 cells there was a loss of cell viability in a dose- and time-dependent manner (Fig. 1a and Supplementary Fig. S1A), which was accompanied with increased cell cytotoxicity (Fig. 1b). The cytotoxic effect of VP was more pronounced in HuH7 cells and coincided with an increase in cells in the sub-G1 phase and a decrease in the G1 phase compared to HepG2 (Supplementary Fig. S1B-C). We next determined if VP is able to potentiate sorafenib (SF), the first-line FDA-approved oral multi-kinase inhibitor used for advanced HCC. SF used as a single agent did not impair tumor cell viability, while in combination with VP there was a synergistic effect in HuH7 cells (coefficient of drug interaction (CDI) = 0.62) and an additive effect in HepG2 (CDI = 0.97). This was accompanied by an increase of cell cytotoxicity (Fig. 1c, d). We next analyzed the effect of SF, VP and their combination on HCC patient-derived HCC tumoroids (PDTs) in vitro. As observed in the HCC cell lines, SF had no effect on PDT survival, however, PDTs exposed to VP displayed a loss of three-dimensional structure (Fig. 1e), and a loss of viability (Fig. 1f). Using an in vivo patient-derived xenograft (PDX) model, we demonstrated that SF in combination with VP markedly reduced tumor growth and progression compared to vehicle and SF alone (Fig. 1g). The antitumor effect of SF and VP was characterized by a significant decrease of proliferating, Ki67-positive cells as well as a significant downregulation of expression of cell-cycle progression genes, cyclin-A2 (CCNA2) and cyclin-B1 (CCNB1; Fig. 1h, i). The anti-angiogenic capability of SF was observed by CD31 staining, in which VP and SF-treated tumors had significantly less intra-tumor vasculature, as well as by downregulation of vascular endothelial growth factor A (VEGF-A) gene expression (Fig. 1h, i). Knowing the effect of VP on the transcriptional complex Yes-associated protein 1 (YAP1) of the Hippo signaling pathway^25^, we confirmed the significant downregulation of YAP1 target genes such as connective tissue growth factor (CTGF) and cysteine-rich angiogenic inducer 61 (CYR61) in vitro for both HCC cell lines, without any regulation when combined with SF (Supplementary Fig. 1D). Interestingly, the apoptotic marker caspase 3 was already less expressed at its pro-caspase levels for both HCC cell lines and for all the VP- and SF/VP-treated animals (Supplementary Fig. S1E-F). Increased levels of cleaved poly ADP ribose polymerase (PARP) were only observed on the most sensitive cell line HuH7 and in SF/VP-treated mice (Supplementary Fig. S1E-F). The inhibitory effect of VP on HCC growth in vivo was confirmed using HepG2 cells in a subcutaneous HCC xenograft mouse model (Supplementary Fig. S2A-C). The effect of SF and VP on angiogenesis was tested using human immortalized microvascular endothelial cells (HMEC-1) in an in vitro tube formation assay (Supplementary Fig. S2D). Interestingly, VP-treated HMEC-1 cells also showed a decrease of newly formed vasculature, however there was no synergistic or additive effect in combination with SF (Supplementary Fig. S2E). These results suggest that VP has a negative effect on the proliferation and progression of HCC and augments the effect of SF in vitro and in vivo.

### VP induces lysosomal membrane permeabilization due to the alkalinization of lysosomal pH

**Fig. 2:**
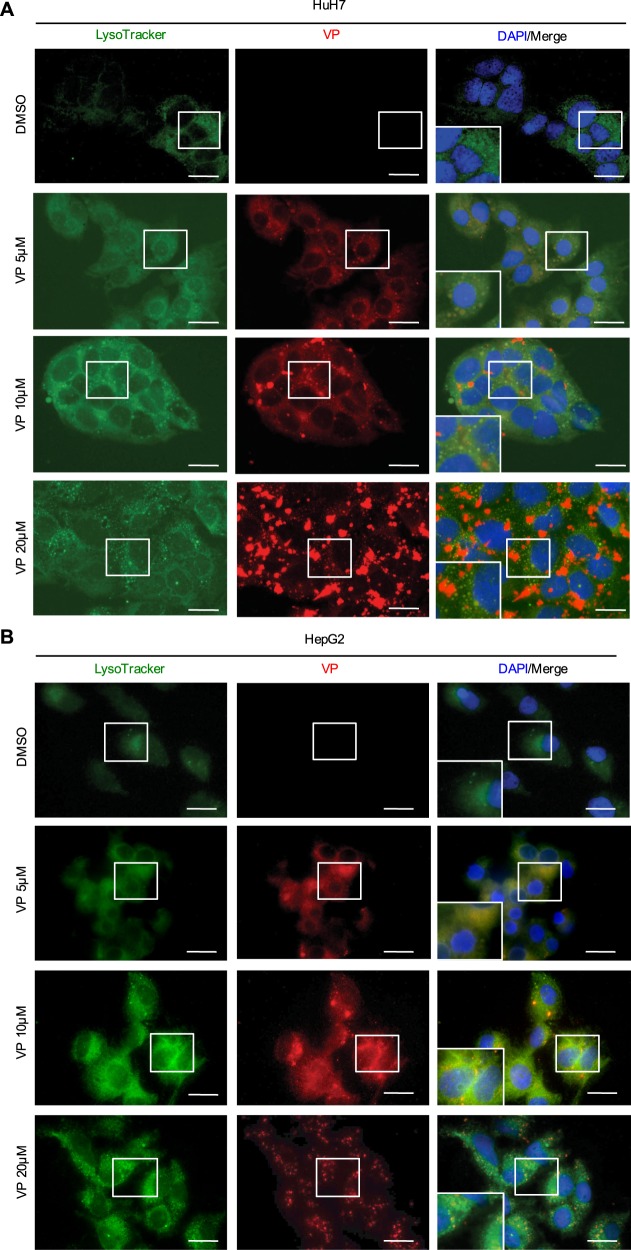
VP targets the lysosomal compartment. **a** Representative live HuH7 and **b** HepG2 cell-images showing the co-localization of the VP autofluorescence after 24 h in the Cy5 channel and Lysotracker staining (1 h, 100 nM) in the Cy3 channel. Nuclei were stained with DAPI. Scale bar 25 μm

**Fig. 3:**
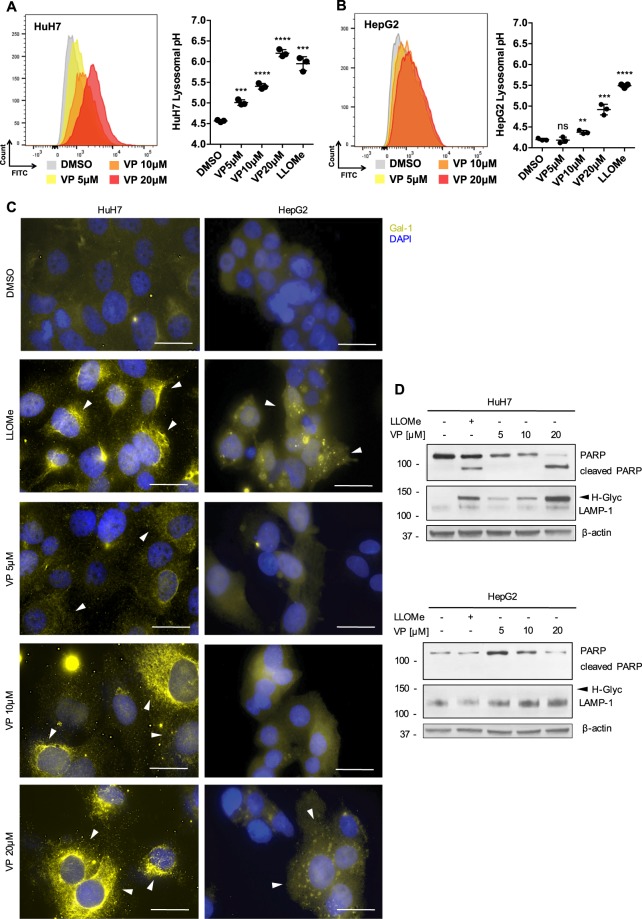
VP-induced lysosomal pH alkalinization induces LMP. **a** Illustrative histograms from flow cytometry showing the shifted FITC-dextran emission wavelength after different concentrations of VP (24 h), LLOMe (6 h, 2.5 mM) and DMSO treatment on HuH7 and **b** HepG2. The graphs show the results from lysosomal pH measurement on HuH7 and HepG2, *n* = 3. **c** Representative LMP galectin-1 staining (white arrows) in HuH7 and HepG2 upon VP (24 h) and the positive control LLOMe (6 h, 2.5 mM) treatment. Nuclei were stained with DAPI. Scale bar 50 μm. **d** Immunoblots for PARP, LAMP-1 and β-actin in HuH7 and HepG2 cells after VP (24 h) and LLOMe (6 h, 2.5 mM) treatment. *P* values < 0.05 were considered statistically significant and are indicated as follows: *P < 0.05; **P < 0.01; ***P < 0.001; ****P < 0.0001; ns, not significant

VP is a chlorinated benzoporphyrin derivative monoacid ring which contains a photoactive ring that is auto fluorescent in the far-red spectrum (660–780 nm)^21^. Interestingly, we found a cytoplasmic co-localization of VP with LysoTracker-stained lysosomes, which then led to an increase of their number and an alteration of their shape in both HCC cell lines (Fig. 2a, b and Supplementary Fig. S3A). VP induced the alkalinization of the lysosomal lumen pH in a dose-dependent manner, similar to that observed for the lysosome-specific membrane damage inducer L-leucyl-L-leucine methyl ester (LLOMe; Fig. 3a, b). The alkalinization of the lysosomal lumen leads to pH-driven instability, which ultimately results in lysosomal membrane permeabilization (LMP)^33,35^. Galectin-1 (gal-1) localization changed from a diffuse to a punctate pattern in the cytoplasm, confirming LMP after LLOMe-induced lysosomal pH alkalinization (Fig. 3c). A similar gal-1 punctate staining pattern was observed in cells treated with VP for both HCC cell lines in a dose-dependent manner, with this more pronounced in HuH7 than HepG2 cells (Fig. 3c). As a marker for lysosomal instability, the expression of lysosomal-associated membrane protein 1 (LAMP-1) was assessed and was found to be hyperglycosylated (H-Glyc) in HuH7 cells treated with LLOMe and VP (Fig. 3d). While, in HepG2 cells, VP induced a dose-dependent increase of LAMP-1 (Fig. 3d). VP- and LLOMe-induced toxicity of lysosomes was also accompanied by increased levels of cleaved PARP, particularly in HuH7 (Fig. 3d). This observation supports the greater cytotoxicity observed in HuH7 cells compared to HepG2 cells (Fig. 1a, b), suggesting a caspase-independent necrotic process after VP treatment (Supplementary Fig. S1E). These results indicate that VP is a lysosomotropic compound and induces lysosomal membranes permeabilization after the alkalinization of intraluminal pH in HCC cell lines.

### VP-induced LMP provokes the dysregulation of intracellular catabolic mechanisms, finally leading to a proteotoxic effect

**Fig. 4:**
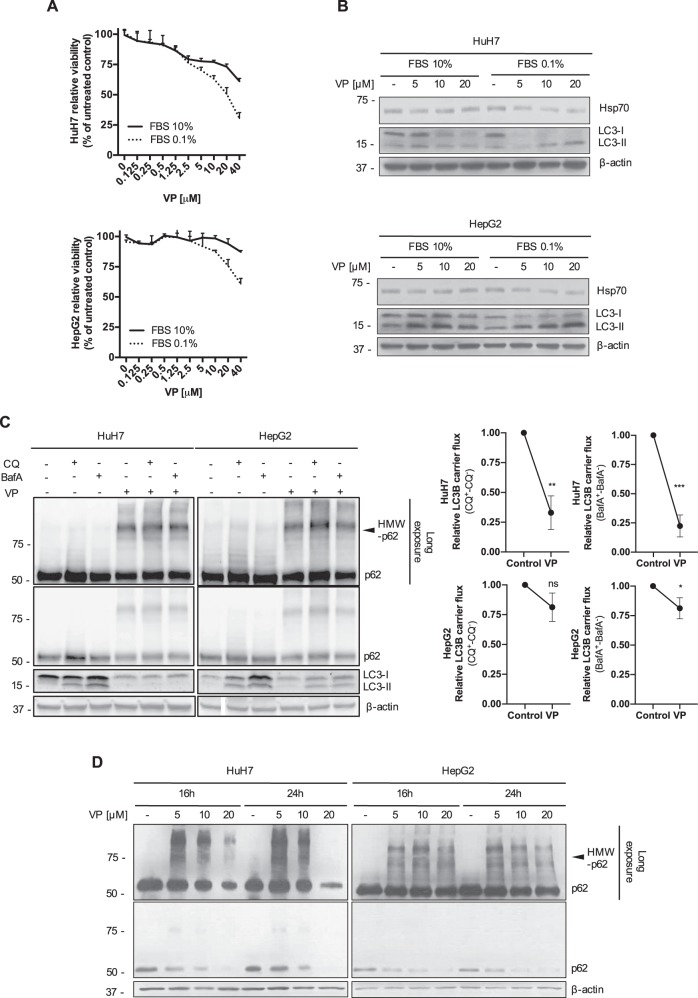
VP-induced dysregulation of the intracellular catabolic machinery provokes a broad tumor-specific proteotoxic effect. **a** Mean ± SD relative viability of HuH7 and HepG2 cells was analyzed after 24 h with various concentrations of verteporfin (VP) treatment under normal (complete medium supplemented with 10% of fetal-bovine serum- FBS) and starving culture conditions (complete medium + 0.1%FBS) and values were normalized to vehicle-treated cells, *n* = 3. **b** Immunoblots for Hsp70, LC3B and β-actin in HuH7 and HepG2 cells after different concentrations of VP (24 h) under normal (complete medium + 10% FBS) and starving culture conditions (complete medium + 0.1%FBS). **c** HuH7 and HepG2 cells were treated with VP (20 μM, 24 h) ±chloroquine (CQ - 20 μM, 4 h) or Bafilomycin A1 (BafA - 100 nM, 4 h) under starving culture conditions (complete medium + 0.1%FBS) before western blot analysis for p62 and LC3B was performed. β-actin served as a loading control. LC3B carrier flux data were generated by normalizing the LC3B-II levels to β-actin followed by substraction of the BafA/CQ untreated sample from its respective BafA/CQ stimulated condition. Flux under control condition was set to 1. **d** Representative immunoblots for p62 and β-actin in HuH7 and HepG2 cells after VP (16 and 24 h) treatment. *P* values < 0.05 were considered statistically significant and are indicated as follows: *P < 0.05; **P < 0.01; ***P < 0.001; ****P < 0.0001; ns, not significant

Having observed an induction of LMP by VP, we investigated if its cytotoxic effect was enhanced by serum deprivation, where tumor cells rely more on intracellular catabolism^36^. Under serum deprived conditions, there was a greater loss of cell viability in a VP dose-dependent manner compared to normal culture conditions in both HCC cell lines (Fig. 4a). Under reduced serum culture conditions, VP treatment induced a dose-dependent decrease in expression of heat shock protein (Hsp70), an evolutionarily conserved molecular chaperone that inhibits LMP (Fig. 4b and Supplementary Fig. S3B), thereby confirming a broad lysosomal compartment instability^37^. Moreover, VP-induced LMP led to a decreased expression of LC3-I on HuH7 compared to HepG2, where instead an accumulation of mature autophagosomes (LC3-II) was mainly detected (Fig. 4b and Supplementary Fig. S3B). To investigate the effect of VP on the autophagic flux, we used two late-stage autophagy inhibitors, chloroquine (CQ) and bafilomycin A1 (BafA). We found that the combination of CQ and BafA with VP under serum deprived conditions resulted in a decreased level of LC3-II accumulation in both cell lines compared to CQ and BafA alone, suggesting that VP reduces autophagic flux (Fig. 4c). On the basis of the known localization of WIPI-1 on the membrane of phagophores^38^, we found a decreased expression of GFP-WIPI-1 cytoplasmic puncta in both cell lines (Supplementary Fig. S3C), confirming that VP reduces autophagy. Interestingly, we confirmed the VP-induced impairment of high–molecular weight oligomerized proteins bound to the sequestrome/cargo protein p62 (HMW-p62) clearance^39^, which significantly accumulate after VP treatment (Fig. 4d), leading to proteotoxicity, autophagic flux interference and LMP. These results suggest that VP-induced LMP seems to be tumor-specific. In malignant cells these phenomena provoke an important catabolic dysregulation, which finally leads to an unsolvable intracellular proteotoxicity.

### The tumor-specificity of VP-induced lysosomal compartment dysregulation, autophagic flux blockade and proteotoxicity

**Fig. 5:**
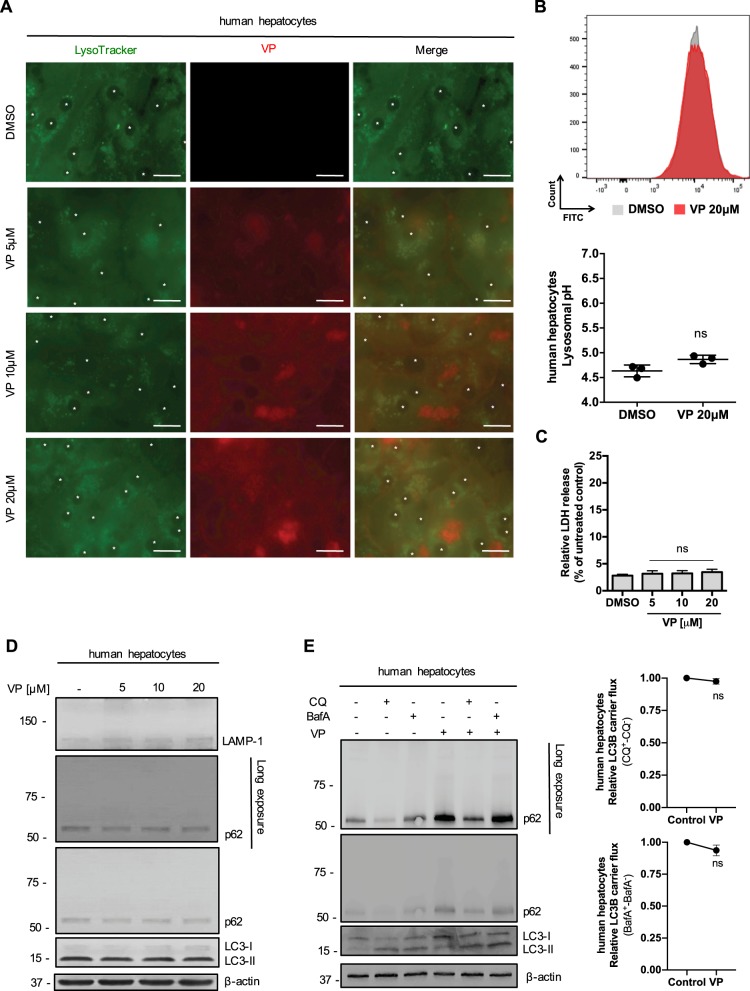
The cytotoxicity and proteotoxicity of VP is tumor-specific. **a** Isolated primary human hepatocytes live imaging showing the co-localization of VP autofluorescence after 24 h in the Cy5 channel and Lysotracker staining (1 h, 100 nM) in the Cy3 channel. Nuclei are marked with “ *”. Scale bar 25 μm. **b** Illustrative histograms from flow cytometry showing FITC-dextran emission wavelength after VP and DMSO 24 h treatment on primary human hepatocytes. The graph shows the results from lysosomal pH measurement. *n* = 3. **c** Mean ± SD relative LDH release in primary human hepatocytes exposed to increasing concentrations of VP and DMSO for 24 h. Values were normalized to untreated primary human hepatocytes. *n* = 3. **d** Immunoblots for LAMP-1, p62, LC3B and β-actin in isolated primary human hepatocytes after different concentrations of VP (24 h) treatment. **e** Primary human isolated hepatocytes were treated with VP (20 μM, 24 h) ±chloroquine (CQ - 20 μM, 4 h) or Bafilomycin A1 (BafA – 100 nM, 4 h) before western blot analysis for p62 and LC3B was performed. β-actin served as a loading control. LC3B carrier flux data were generated by normalizing the LC3B-II levels to β-actin followed by substraction of the BafA/CQ untreated sample from its respective BafA/CQ stimulated condition. Flux under control condition was set to 1. *P* values < 0.05 were considered statistically significant and are indicated as follows: *P < 0.05; **P < 0.01; ***P < 0.001; ****P < 0.0001; ns, not significant

We confirmed the high tropism of VP for the lysosomal compartment also on primary human hepatocytes (Fig. 5a). An increased number of VP-loaded lysosomes was observed only for the highest dose of VP-treated hepatocytes after 24 h (Fig. 5a). An increase in the pH of the lysosomes towards alkaline values was not observed (Fig. 5b). The VP-induced cytotoxic effect previously observed was specific to HCC cells as there was no LDH released from primary isolated human hepatocytes treated with VP (Fig. 5c). No proteotoxic effect on primary isolated human hepatocytes was observed after VP treatment, with no changes in LAMP-1 glycosylation, LC3B expression compared to the control (Fig. 5d). The autophagic flux progression in the presence of CQ and BafA was not modulated in the presence of VP (Fig. 5e), as was observed for HCC cell lines (Fig. 4c). An increase of p62 was observed with VP alone and in combination with the two autophagy inhibitors without any HMW-p62 accumulation (Fig. 5e). These results confirm VP as a lysosomotropic compound, able to induce a tumor-specific LMP after the alkalinization of intraluminal pH.

### Sorafenib treatment leads to an accumulation of lysosomes in vitro and in vivo

**Fig. 6:**
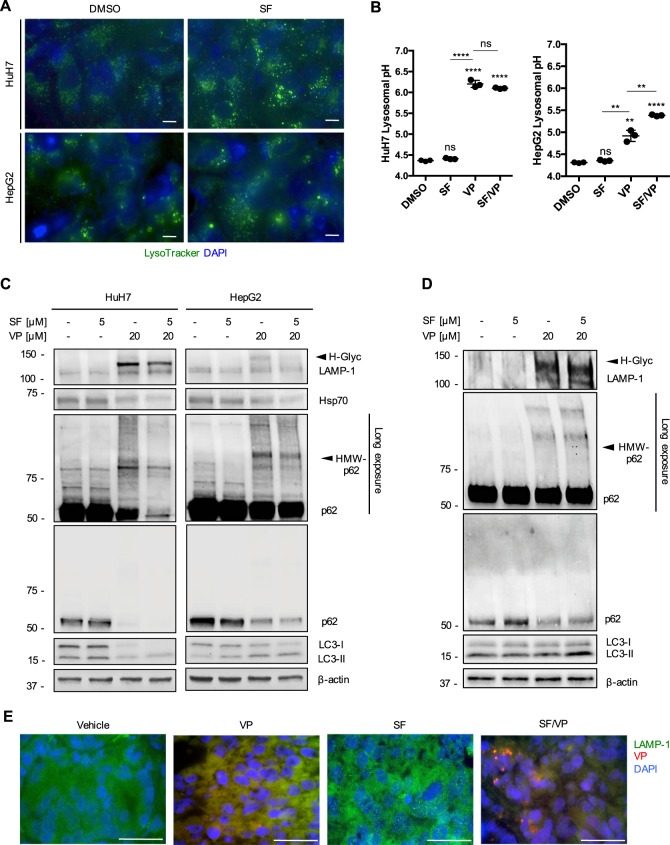
SF induces an accumulation of lysosomes in vitro and in vivo. **a** Representative HCC cell-images live-stained with Lysotracker (1 h, 100 nM) in the Cy3 channel, treated with sorafenib (SF, 5 μM) for 24 h. Nuclei were stained with DAPI. Scale bar 25 μm. **b** Mean ± SD of lysosomal pH values measured by flow cytometry in HuH7 and HepG2 pre-incubated in culture medium supplemented with FITC-dextran (0.1 mg/mL, 72 h) after treatment with SF (5 μM), VP (20 μM) and both drugs combined together (VP/SF) for 24 h. *n* = 3. **c** Representative immunoblots for LAMP-1, Hsp70, p62, LC3B, and β-actin in HuH7 and HepG2 cells after VP (20 μM, 24 h), SF (5 μM, 24 h) treatment and both compounds combined together. **d** Representative immunoblots for LAMP-1, p62, LC3, and β-actin in HCC patient-derived tumoroids (PDT) after VP (20 μM, 24 h), SF (5 μM, 24 h) treatment and both compounds combined together, *n* ≥ 3. **e** Illustrative immunofluorescence images on cryosections of LAMP-1 and VP autofluorescence (Cy5 channel) from HCC patient-derived xenografts after 14 days of treatment with vehicle, VP, SF and SF/VP. Nuclei were stained with DAPI. Scale bar 50 μm. *P* values < 0.05 were considered statistically significant and are indicated as follows: *P < 0.05; **P < 0.01; ***P < 0.001; ****P < 0.0001; ns, not significant

According to the Drug Bank database that SF has similar chemical properties to the other TKIs (acid dissociation constant - pKa and lipophilicity coefficient - logP)^5^, we therefore tested the affinity of SF for the lysosomal compartment. We found a cytoplasmic increase of LysoTracker-stained lysosomes in HuH7 and HepG2 cells after SF treatment (Fig. 6a and Supplementary Fig. S4A). The acidic intraluminal pH was not altered with SF, while VP alone or in combination with SF resulted in a significant alkalinization of lysosomal pH (Fig. 6b). SF alone did not alter the expression of LAMP-1 or Hsp70 compared to the control-treated cells (Fig. 6c). A lower glycosylation of LAMP-1 and a lower expression of Hsp70 were observed for both HCC cell lines treated with SF/VP, suggesting that SF seems to be passively sequestered into lysosomes, which then are permeabilized by VP (Fig. 6c). A slight increased lipidation rate of the autophagic protein LC3B was detected in both HCC cell lines after SF treatment, confirming the known induction of autophagy compared to the controls (Fig. 6c and Supplementary Fig. S4B)^19^. After SF/VP treatment, the progression of autophagy was almost completely blocked in both HCC cell lines (Fig. 6c and Supplementary Fig. S4B). Looking at autophagic flux modulation, we found that the combination of CQ and BafA with SF resulted in a significant induction of autophagy (Supplementary Fig. 4C-D). The SF-induced autophagic flux was significantly impaired in combination with VP in both HCC cell lines (Supplementary Fig. 4C-D). No relevant regulation of p62 expression was observed for SF alone or in combination with the two autophagy inhibitors, while proteotoxicity was detected only in the presence of VP (Fig. 6c and Supplementary Fig 4C). We also analyzed the expression of the transcription factor EB (TFEB) target genes in the coordinated lysosomal expression and regulation (CLEAR) network^40^ (Supplementary Fig. 4E). TFEB was significantly upregulated in SF-treated HCC cell lines, but were downregulated with VP and much more in SF/VP-treated cells (Supplementary Fig. 4E). LAMP-1 expression was upregulated in SF-treated cells, while significantly downregulated in the presence of VP for the most sensitive cell line HuH7 (Supplementary Fig. 4E). Interestingly, by looking at the expression of genes involved in lysosomal hydrolase (cathepsin D – CTSD) and lysosomal acidification (ATPase H^+^ transporting V0 subunit D2 – ATP6V0D2; Chloride channel 7 – CLCN7) we found a SF-induced upregulation (especially in HuH7 cells). In contrast, they were significantly downregulated with VP as a single treatment and even more in combination with SF (Supplementary Fig. 4E). Similar results were also obtained for PDTs, where SF/VP treatment led to a higher glycosylation status of LAMP-1, an impairment of autophagy progression (higher LC3-II accumulation) and an increased proteotoxic effect (HMW-p62; Fig. 6d). Similar to the in vitro models, an accumulation of LAMP-1-stained lysosomes was observed in PDXs with SF treatment compared to vehicle-treated mice (Fig. 6e). VP-autofluorescence was also detectable in the lysosomal compartment within the PDX tumor cryosections, where indeed the SF/VP-treated animals showed a decrease of intra-tumoral accumulation of LAMP-1 (Fig. 6e). Given its importance in cell growth and metabolism in HCC^41^, we analyzed the target of rapamycin (TOR) and the status of its downstream targets P70-S6 Kinase 1 (S6K) and the eukaryotic translation initiation factor 4E (eIF4E)-binding protein 1 (4E-BP1). We found a significantly decreased expression of mTOR and phospho-mTOR (p-mTOR) proteins after VP treatment either alone or in combination with SF for both HCC cell lines (Supplementary Fig. 5A-B). Same significant trend was observed for the expression of the phosphorylated forms of S6 kinase and 4E-BP1 in the presence of VP, especially in HuH7 cells compared to HepG2 (Supplementary Fig. 5A-B). VP-induced lysosomal compartment instability and dysregulation of the autophagic machinery observed in vitro were confirmed in the PDX model, with an increased glycosylation rate of LAMP-1 and HMW-p62 proteotoxic aggregates, accompanied by a consequent inverted expression of LC3B (Supplementary Fig. S5C). We showed that SF is lysosomotropic, leading us to hypothesize that it could also be sequestered in the lumen of lysosomes. Therefore VP-induced lysosomal pH alkalinization, LMP and proteotoxicity are the main molecular events of the interaction with SF.

### RAS oncoprotein status seems to modulate the sensitivity to VP-induced toxicity and lysosomal pH alkalinization in HCC cell lines

**Fig. 7:**
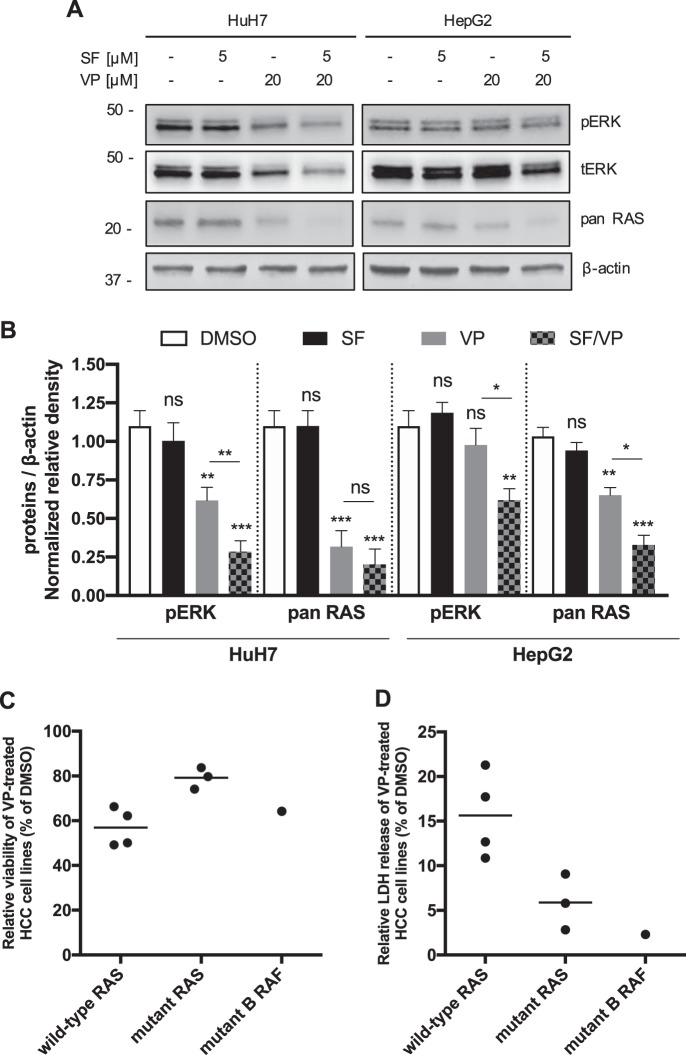
RAS status modulates cell sensitivity during lysosomal pH alkalinization induced by VP. **a** Representative immunoblots and quantification, **b** for pERK, tERK, pan RAS and β-actin in HuH7 and HepG2 cells after VP (20 μM, 24 h), SF (5 μM, 24 h) treatment and both compounds combined together. **c** Mean of relative viability and **d** LDH release of a panel of eight different HCC cell lines was analyzed after 72 h of verteporfin (VP – 20 μM) treatment and values were normalized to vehicle-treated (DMSO) cells. Both box plot graphs show the mean values from the eight HCC cell lines grouped by their specific RAS and RAF status. *P* values < 0.05 were considered statistically significant and are indicated as follows: *P < 0.05; **P < 0.01; ***P < 0.001; ****P < 0.0001; ns, not significant

The intracellular molecular targets of SF on epithelial tumor cells are v-raf murine sarcoma viral oncogene homolog B1 (B-RAF) and murine leukemia viral oncogene homolog 1 (Raf1)^42^. We did not observe a decrease of ERK phosphorylation (pERK) in both HCC cell lines after SF treatment (Fig. 7a, b). We found that only after VP-induced LMP there was a significant decrease of total ERK (tERK) and pERK protein expression either alone (HuH7) or in combination with SF for both HCC cell lines (Fig. 7a, b). Keeping in mind the marked and persistent different responsiveness observed in HuH7 and HepG2 cells and the different expression of pERK after VP, SF and SF/VP treatment, we attempted to investigate the activity of the MAPK pathway signaling nodes located upstream of ERK, by assessing the expression of the small GTPase human Rat Sarcoma (RAS) oncoprotein. Indeed, VP decreased the expression of RAS, which was even more pronounced in combination with SF (Fig. 7a, b). Due to the fact that cancer cells often have multiple genetic aberrations leading to differences in drug sensitivity^43,44^ and having observed a significant LMP-induced reduction of RAS oncoprotein expression, we then tested the efficacy of VP on a panel of seven distinct HCC cell lines differing in their RAS status (wild-type and mutated) and mutant B-RAF (SK-Hep-1 cell line). The negative effect on cell viability as well as the cytotoxic LDH release induced by VP was stronger on wild-type RAS HCC cell lines than the mutant RAS and B-RAF cell lines (Fig. 7c, d and Supplementary Fig. S5A-B). These results show that SF-induced lysosomes accumulation correlates with a decreased availability and efficacy at its intracellular target site. It might suggest an important role of the RAS-status in the context of lysosomal compartment stability after LMP in HCC cell lines.

## Discussion

**Fig. 8:**
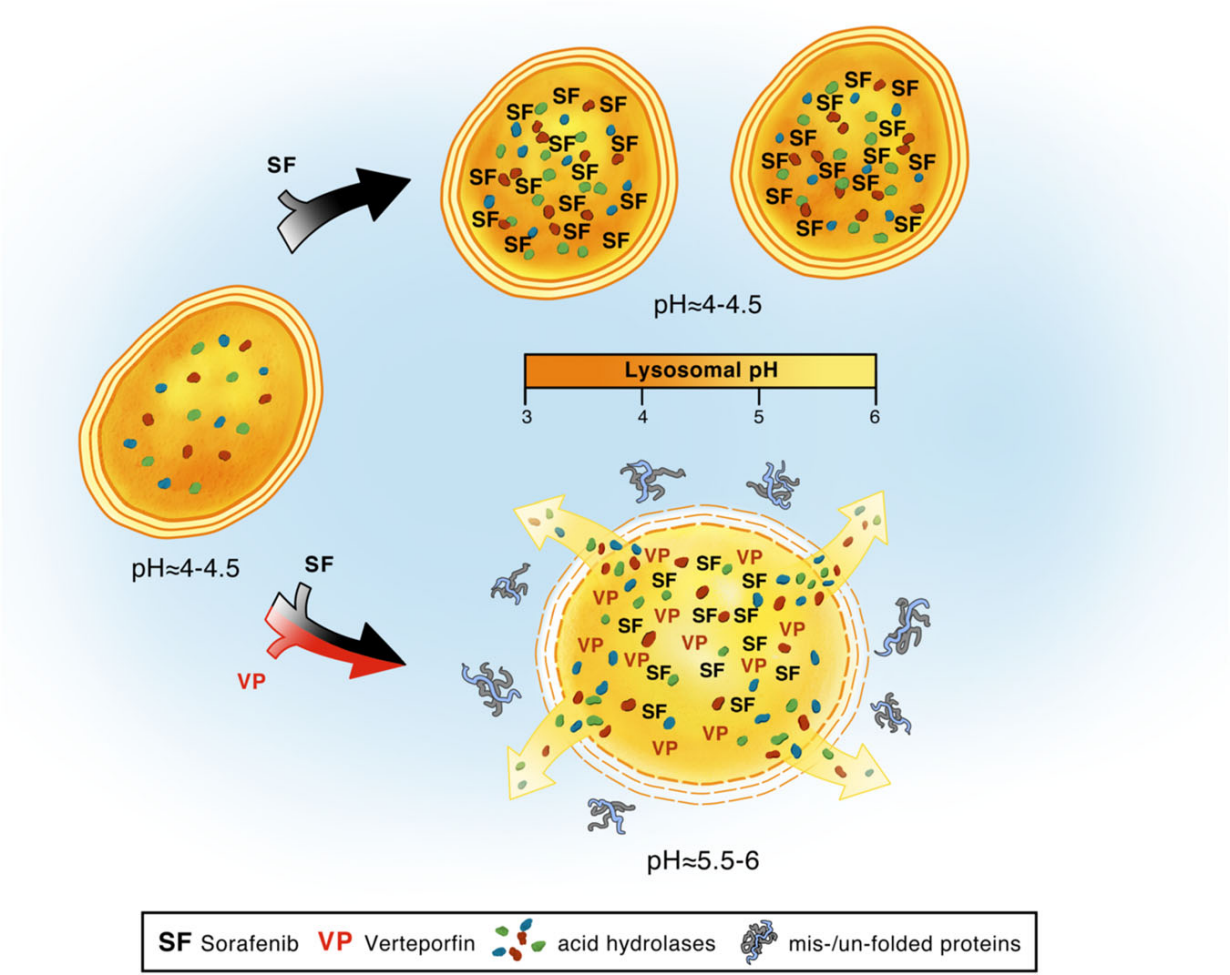
Proposed model of synergic interaction of sorafenib and verteporfin. Sorafenib treatment induces an accumulation of lysosomes and is passively sequestered within their acid lumen. These phenomena reduce SF intracellular concentration and distribution at the target site. The lysosomotropic compound VP, either alone or in combination with SF, accumulates in lysosomes, actively induces an alkalinization of intraluminal pH which then leads to a diffuse lysosomal membrane permeabilization. VP-induced lysosomal compartment instability leads to a broad autophagic flux dysregulation and consequent proteotoxic effect

In this study we have revealed an alternative mechanism of action of VP in the context of cancer therapy, in which it is sequestered in lysosomes and thereby alters lysosomal function. VP, similarly to the well-described lysosome-specific membrane damage inducer LLOMe, triggers an alkalinization of lysosomal pH and a consequent induction of LMP^45,46^. The sequestering of VP in the lysosomal compartment of HCC cells results in cell toxicity, which was not observed in non-malignant liver cells. Hepatocyte survival was not affected by VP, whereas malignant cells underwent LMP, with an increased proteotoxicity mainly caused by lysosomal pH alkalinization and consequent non-functional intracellular catabolic mechanisms. Previous studies have shown differences in lysosomal compartment stability between normal and malignant cells under normoxic or hypoxic and nutrient-deprived or nutrient-rich environments^47^. While normal tissue has a lower grade of stress induced by proteosomal and autophagic recycling processes, tumor cells show a heightened susceptibility upon exposure to lysosomal-mediated cell death agents^24,47^. There are numerous cancer therapeutics that are ultimately sequestered to the lysosome^9,48,49^. Anti-cancer therapy can be diminished or can lead to drug resistance, as the concentration of lysosomotropic compounds is reduced and they are physically unable to reach their molecular target sites^10,50,51^. Based on the predictive mathematical model reported by Trapp et al.^52^, we found that SF is also lysosomotropic. We showed that SF, by inducing a significant increase of lysosomes in vivo and in vitro, could also be sequestered in the lumen of lysosomes. The lysosomotropism of SF did not alter all the lysosomal stability markers (intraluminal pH, LAMP-1, and Hsp70), compared to what we observed in cells treated with VP. Here we propose to combine SF with VP in order to elicit a dual inhibitory effect on autophagy and lysosomal compartment. After the passive lysosomal sequestration of SF and a significant upregulation of TFEB and LAMP-1 genes, VP induces an alkalinization of intraluminal lysosomal pH and LMP with a significant downregulation of CLEAR genes involved in lysosomal hydrolase and acidification. In our in vitro and in vivo models, we confirmed that SF enhanced autophagy compared to the controls^19^, while conversely it was blocked in combination with VP. A greater accumulation of lysosomes was also observed in PDXs after SF treatment, which was ultimately abrogated by VP, accompanied by an increase of the glycosylation rate of LAMP-1. When HuH7 or HepG2 (both B-RAF wild type) were treated with SF, we did not observe a decrease in the phosphorylation of the downstream ERK1/2 kinase, which may be due to its accumulation in the acidic lumen of lysosomes and a drastically decreased concentration at its target site. An overwhelmed lysosomal compartment stability has been described as an uncontrolled process, where the release of acidic content and proteases into the cytoplasm after LMP can lead to necrosis without any caspase activation^53^. Similarly to previous observations^54^, we showed that the VP-induced cleavage of PARP in in vitro and in vivo models, suggests a caspase-independent necrotic process due to the release of the acidic content and proteases from the permeabilized lysosomal lumens. We confirmed that after SF treatment, the viability of HCC cell lines and patient-derived tumoroids was not reduced^34^. Interestingly, we found that in tumor cells treated with VP there was a marked decrease in RAS protein expression, an effect that was even more pronounced in combination with SF. Knowing the peripatetic role of RAS and its physical co-localization with LAMP-1^55,56^, we demonstrated that VP-induced LMP correlates with the proportional proteolysis of RAS in tumor cells. We were also able to show a RAS-dependent sensitivity to LMP, where cells with mutated RAS were less sensitive to VP-induced LMP then the RAS wild-type HCC cell lines. Taken together, our data suggest that the hydrophobic and weak-base tyrosine kinase inhibitor SF seems to be passively sequestered in lysosomes, which then leads to a reduction of its cytoplasmic concentration and therefore access to its target sites. This can result in a loss of tumor cell SF-induced toxicity and induce drug resistance (Fig.8). Instead, VP is also sequestered in the lysosomal lumen, but then it actively induces a high lysosomal compartment destabilization and an autophagy dysregulation. These phenomena are caused by a VP-induced alkalinization of the lysosomal pH, followed by a tumor-specific cytotoxic accumulation of misfolded proteins, which ultimately potentiate the anti-tumor effect of SF (Fig. 8). The VP-induced tumor-selective proteotoxic effect and LMP, may prove critical in treating tumors, whose therapeutic options are limited. Used in combination with SF, VP might help to overcome chemo-insensitivity caused by passive lysosomal sequestration of anti-cancer drugs and ameliorate HCC-patient outcomes.

## Supplementary information

Supplementary Figure captions

Supplementary Figure 1

Supplementary Figure 2

Supplementary Figure 3

Supplementary Figure 4

Supplementary Figure 5

Supplementary Figure 6
