# Supplementary material for: Verteporfin-induced lysosomal compartment dysregulation potentiates the effect of sorafenib in hepatocellular carcinoma
Source: Cell Death Dis. 2019 Oct 3;10(10):749. doi: 10.1038/s41419-019-1989-z (PMC6776510; doi:10.1038/s41419-019-1989-z)
Supplement: Supplementary file 1 — Supplementary Figure captions [file 41419_2019_1989_MOESM1_ESM.pdf]

Supplementary Figure 1: (A) Mean ± SD of HuH7 and HepG2 relative viability was analyzed after 72h of VP treatment. Values were normalized to untreated cells, n=3. **(B)** Flow-cytometry analysis of cell-cycle distribution in HuH7 and **(C)** HepG2 cells treated with VP for 24 hours. n=3. **(D)** Real-time qPCR data of YAP-target genes CTGF and CYR61 after VP, SF and VP/SF treated HCC cell lines after 24 h. Values were normalized to the DMSO-treated cells. n=3. **(E)** Immunoblots for PARP, caspase 3 and β-actin in HuH7 and HepG2 cells after VP (20 μM, 24 h), SF (5 μM, 24 h) treatment and both compounds combined together. **(F)** Immunoblots for PARP, caspase 3 and β-actin of total tumor lysate from HCC patient-derived xenografts after 14 days of treatment with vehicle, VP, SF and SF/VP. P values <0.05 were considered statistically significant and are indicated as follows: *, P < 0.05; **, P < 0.01; ***, P < 0.001; ****, P < 0.0001; ns, not significant.

Supplementary Figure 2: (A) Tumor volume of subcutaneous HepG2-injected xenograft mouse model (n=5/group) treated with vehicle, verteporfin (VP- 100g/kg – i.p. every second day), sorafenib (SF - 60g/kg – daily oral gavage) and VP/SF combination for 14 days. **(B)** Immunohistochemistry for Ki67 and CD31 stainings in vehicle, VP, SF and VP/SF treated animals after 14 days. Counterstain: hematoxylin. Scale bar 200μm. The graphs show the mean percentage ± SD of Ki67 positive cells and CD31 positive areas per tumor/mouse (three random fields were used for the analysis per tumor/mouse). **(C)** Real-time qPCR data of cell-cycle progression (CCNA2 and CCNB1) and vascular endothelial growth factor A (VEGF-A) genes after VP, SF and VP/SF treated animals after 14 days. Values were normalized to the vehicle-treated mice. **(D)** Representative bright-field images of Matrigel® - based angiogenesis tube formation assay on human microvascular endothelial cells (HMEC-1) treated with SF (5μM), VP (20μM), both drugs combined together (SF/VP) and vehicle-treated for 12h. Scale bar 200μm. **(E)** Mean ± SD of relative total endothelial tube length analyzed by a semi-automated plug-in for ImageJ software on corresponding Master trees [Gilles Carpentier. Contribution: Angiogenesis Analyzer. ImageJ News, 5 October 2012.], after HMEC-1 cells treatment with SF (5μM), VP (20μM) and both drugs combined together (SF/VP). Values were normalized to vehicle-treated cells for 12h, n=3. P values <0.05 were considered statistically significant and are indicated as follows: *, P < 0.05; **, P < 0.01; ***, P < 0.001; ****, P < 0.0001; ns, not significant.

Supplementary Figure 3: (A) Mean ± SD of fluorescence intensity of LysoTracker staining determined by spectrophotometry on HuH7 and HepG2 cells after 24h of VP treatment, n=6. **(B)** Immunoblots quantification for LC3-I, -II and Hsp70 from Figure 5B. **(C)** Representative confocal images of GFP-WIPI-1 puncta (white arrows) in HuH7 and HepG2 cells after vehicle (DMSO, 24 h) and VP (20 µM, 24 h) treatment and quantification thereof. n=10 (analyzed fields per sample). Nuclei were stained with DAPI. Scale bar 50 µm. P values <0.05 were considered statistically significant and are indicated as follows: *, P < 0.05; **, P < 0.01; ***, P < 0.001; ****, P < 0.0001; ns, not significant.

Supplementary Figure 4: (A) Mean ± SD of fluorescence intensity of LysoTracker staining determined by spectrophotometry on HuH7 and HepG2 cells after 24h of SF treatment, n=6. **(B)** Immunoblots quantification for LC3-I and -II from Figure 6C. **(C)** HuH7 and HepG2 cells were treated with SF (5 µM, 24h) and VP (20 µM, 24h) +/- chloroquine (CQ - 20 µM, 4h) or Bafilomycin A1 (BafA – 100 nM, 4h) before western blot analysis for p62 and LC3B was performed. β-actin served as a loading control. **(D)** LC3B carrier flux data were generated by normalizing the LC3B-II levels to β-actin followed by substraction of the BafA/CQ untreated sample from its respective BafA/CQ stimulated condition. Flux under control condition was set to 1. **(E)** Real-time qPCR data of TFEB, LAMP1, CSTD, CLCN7 and ATP6V0D2 genes after VP, SF and VP/SF treated HCC cell lines after 24 h. Values were normalized to the DMSO-treated cells. n=3. P values <0.05 were considered statistically significant and are indicated as follows: *, P < 0.05; **, P < 0.01; ***, P < 0.001; ****, P < 0.0001; ns, not significant.

Supplementary Figure 5: (A) Representative immunoblots and quantification **(B)** for mTOR, p-mTOR (Ser 2448), S6 kinase, p-S6 kinase, 4E-BP1, p-4E-BP1 and β-actin in HuH7 and HepG2 cells after VP (20 µM, 24 h), SF (5µM, 24h) treatment and both compounds combined together. **(C)** Immunoblots for LAMP-1, p62, LC3B and β-actin of total tumor lysate from HCC patient-derived xenografts after 14 days of treatment with vehicle, VP, SF and SF/VP. P values <0.05 were considered statistically significant and are indicated as follows: *, P < 0.05; **, P < 0.01; ***, P < 0.001; ****, P < 0.0001; ns, not significant.

Supplementary Figure 6: (A) Mean ± SD of relative viability and **(B)** LDH release of a panel of eight different HCC cell lines was analyzed after 72h of verteporfin (VP - 20µM) treatment and values were normalized to vehicle-treated (DMSO) cells. n=3.
