# Supplementary figures and images for: Verteporfin-induced lysosomal compartment dysregulation potentiates the effect of sorafenib in hepatocellular carcinoma
Source: Cell Death Dis. 2019 Oct 3;10(10):749. doi: 10.1038/s41419-019-1989-z (PMC6776510; doi:10.1038/s41419-019-1989-z)

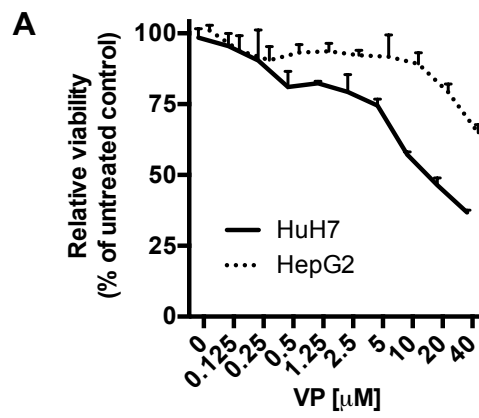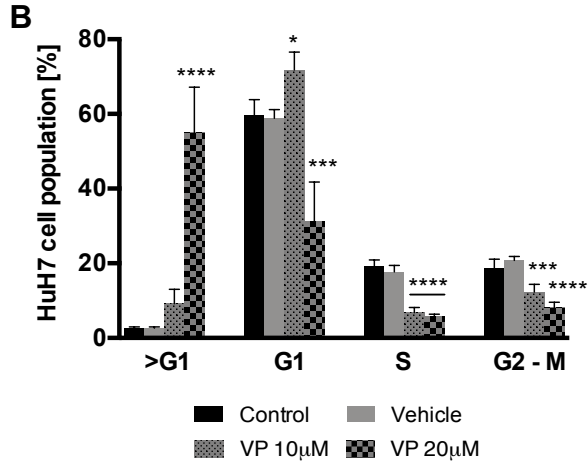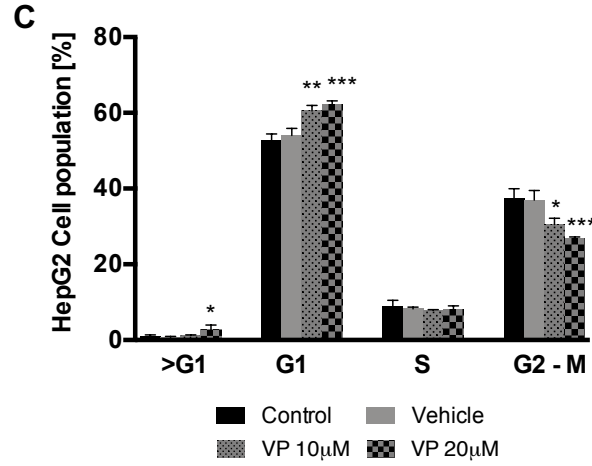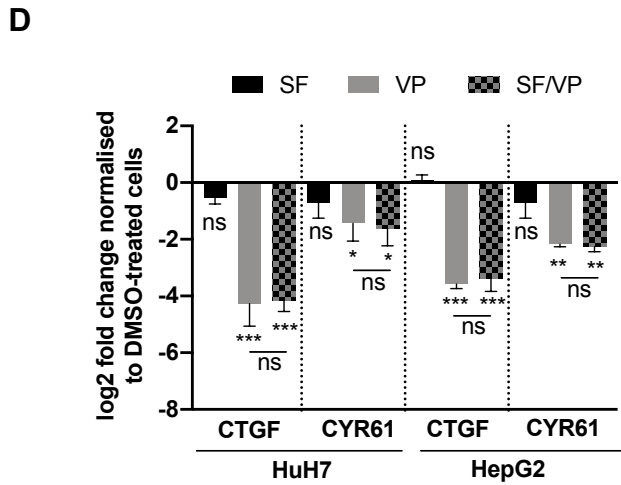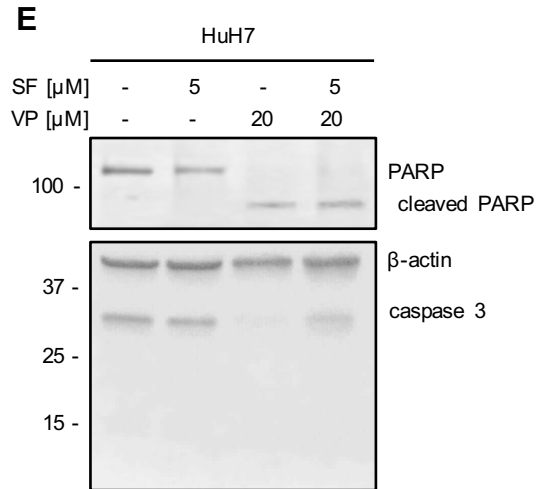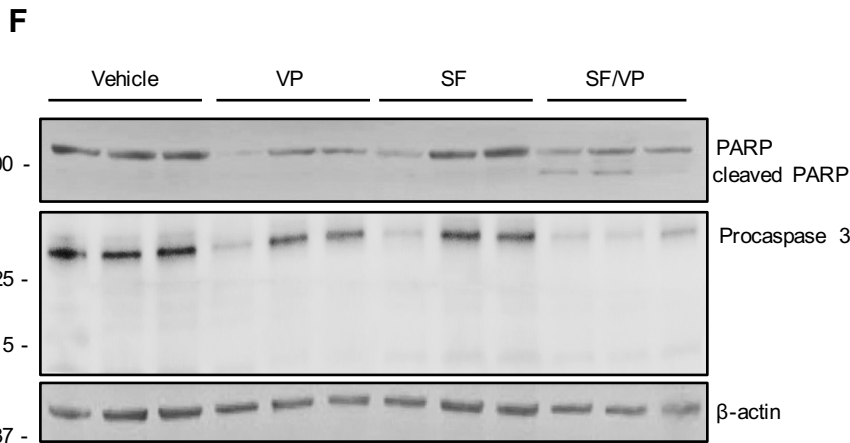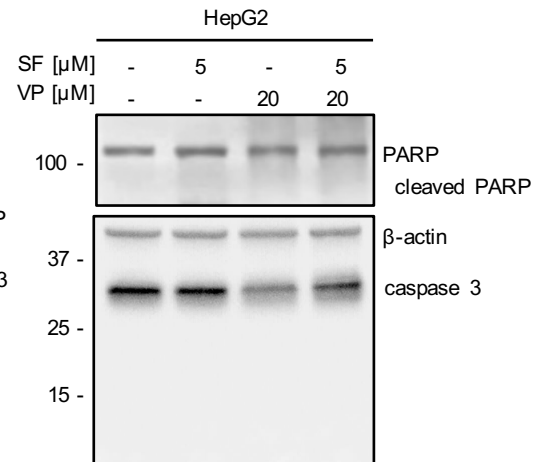

Supplement: Supplementary file 2 — Supplementary Figure 1 [file 41419_2019_1989_MOESM2_ESM.pdf]

**A**

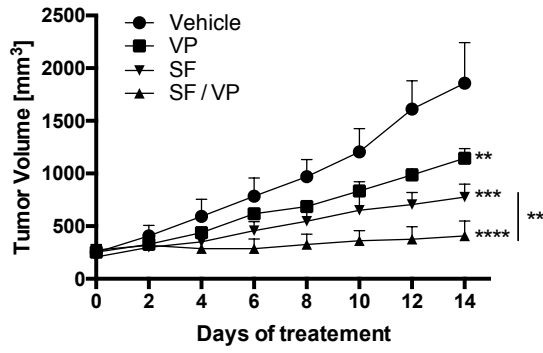

**B**

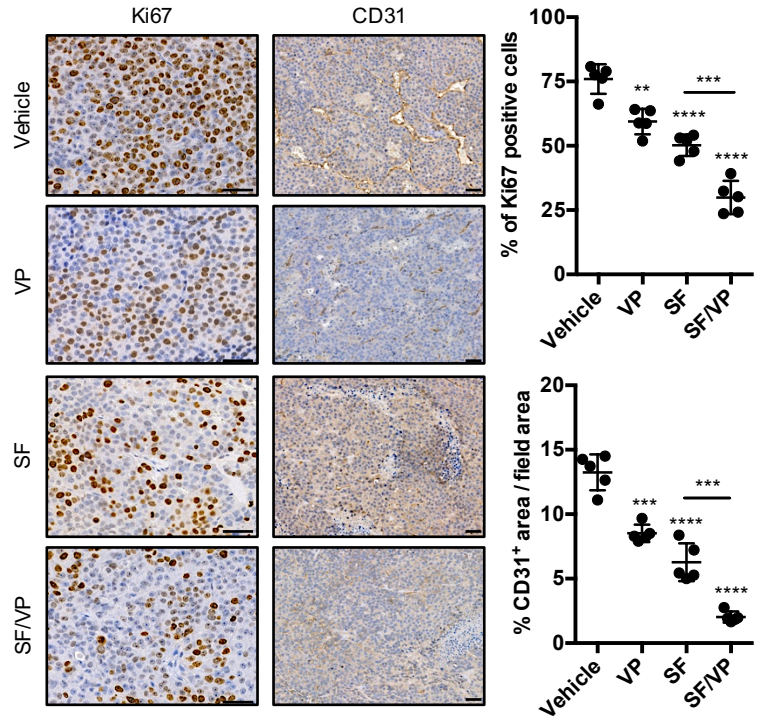

**C**

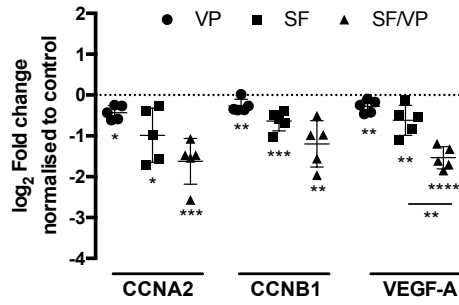

**D**

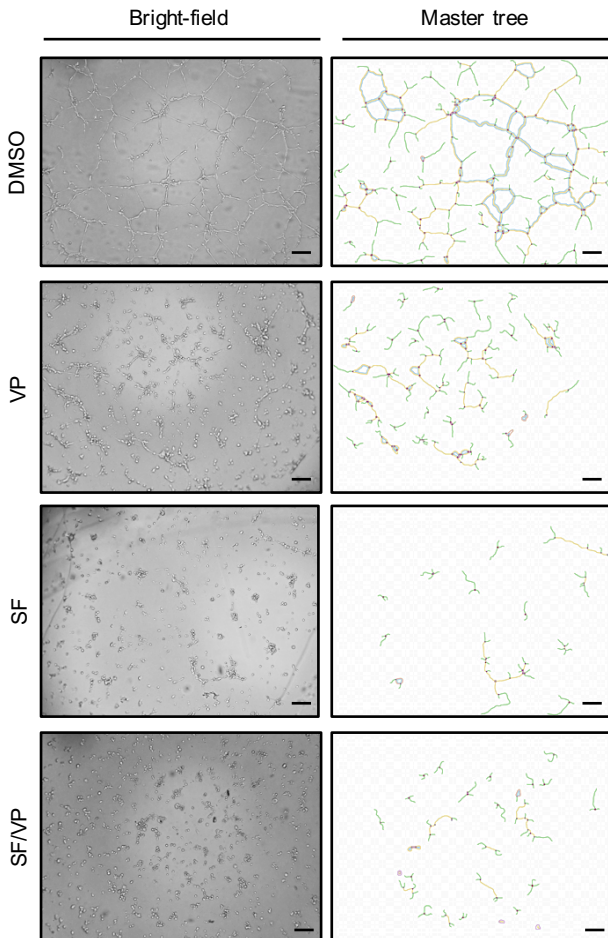

**E**

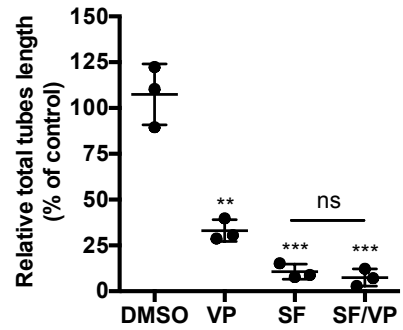

Supplement: Supplementary file 3 — Supplementary Figure 2 [file 41419_2019_1989_MOESM3_ESM.pdf]

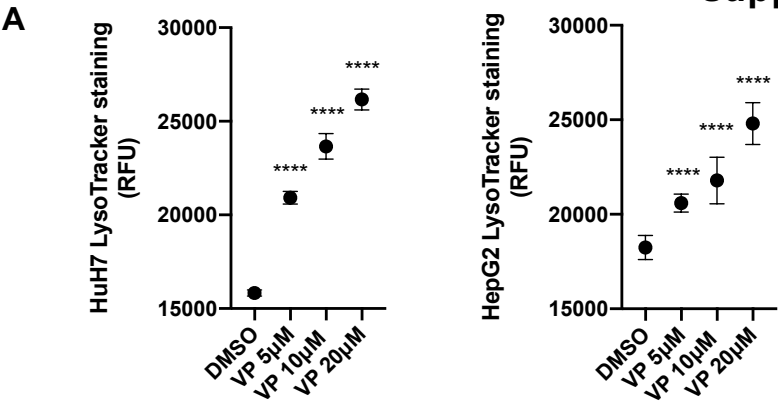

**B**

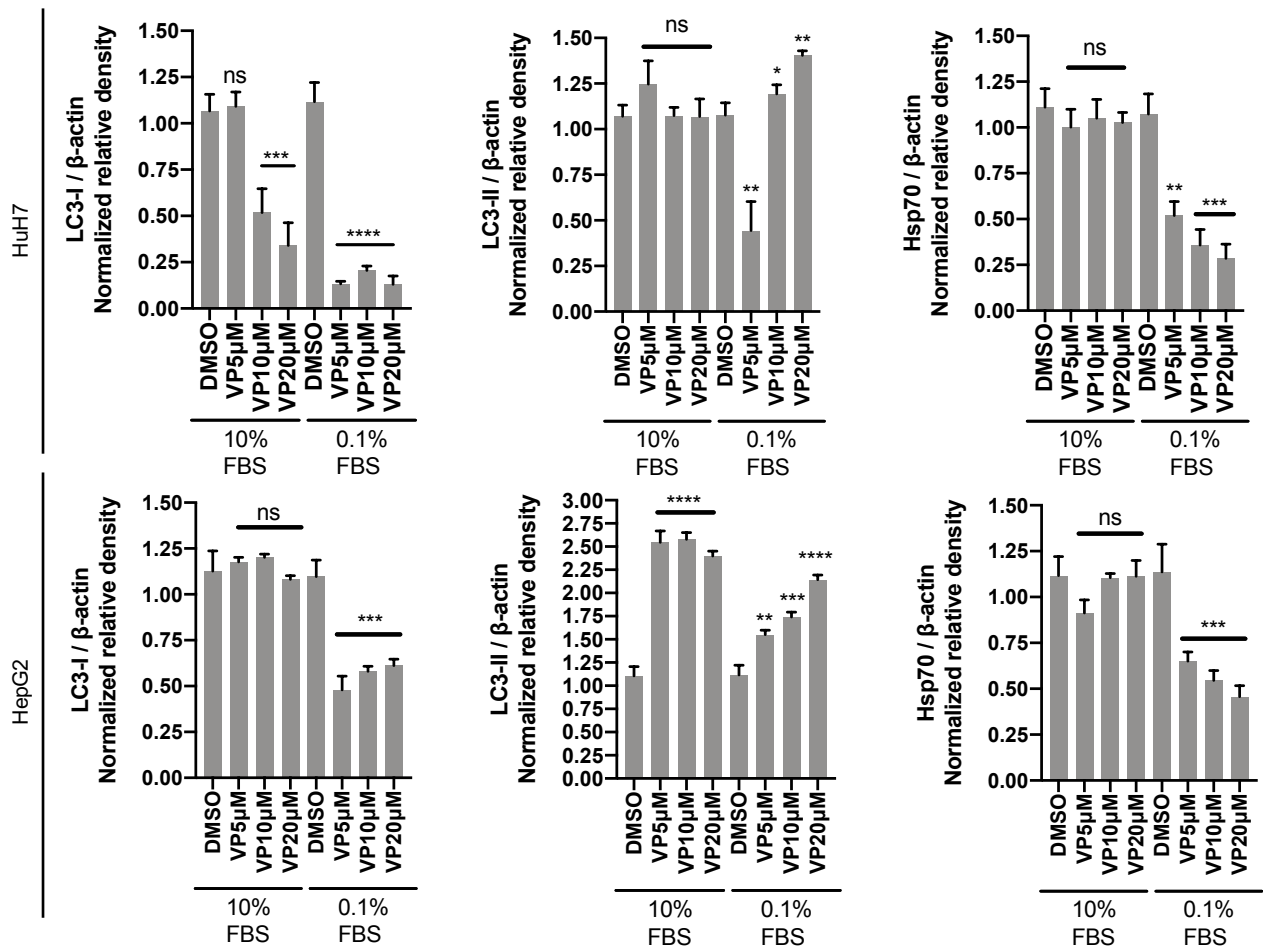

**C**

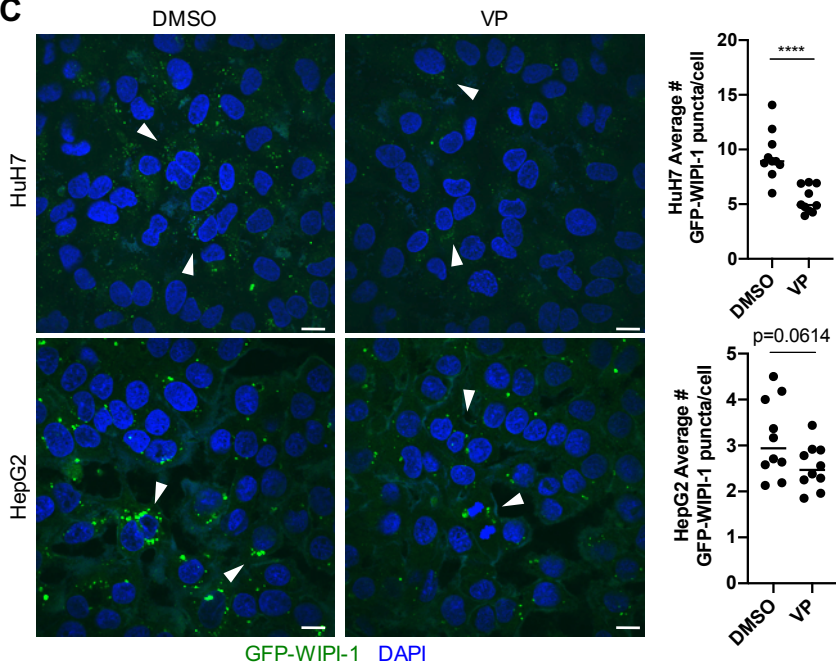

Supplement: Supplementary file 4 — Supplementary Figure 3 [file 41419_2019_1989_MOESM4_ESM.pdf]

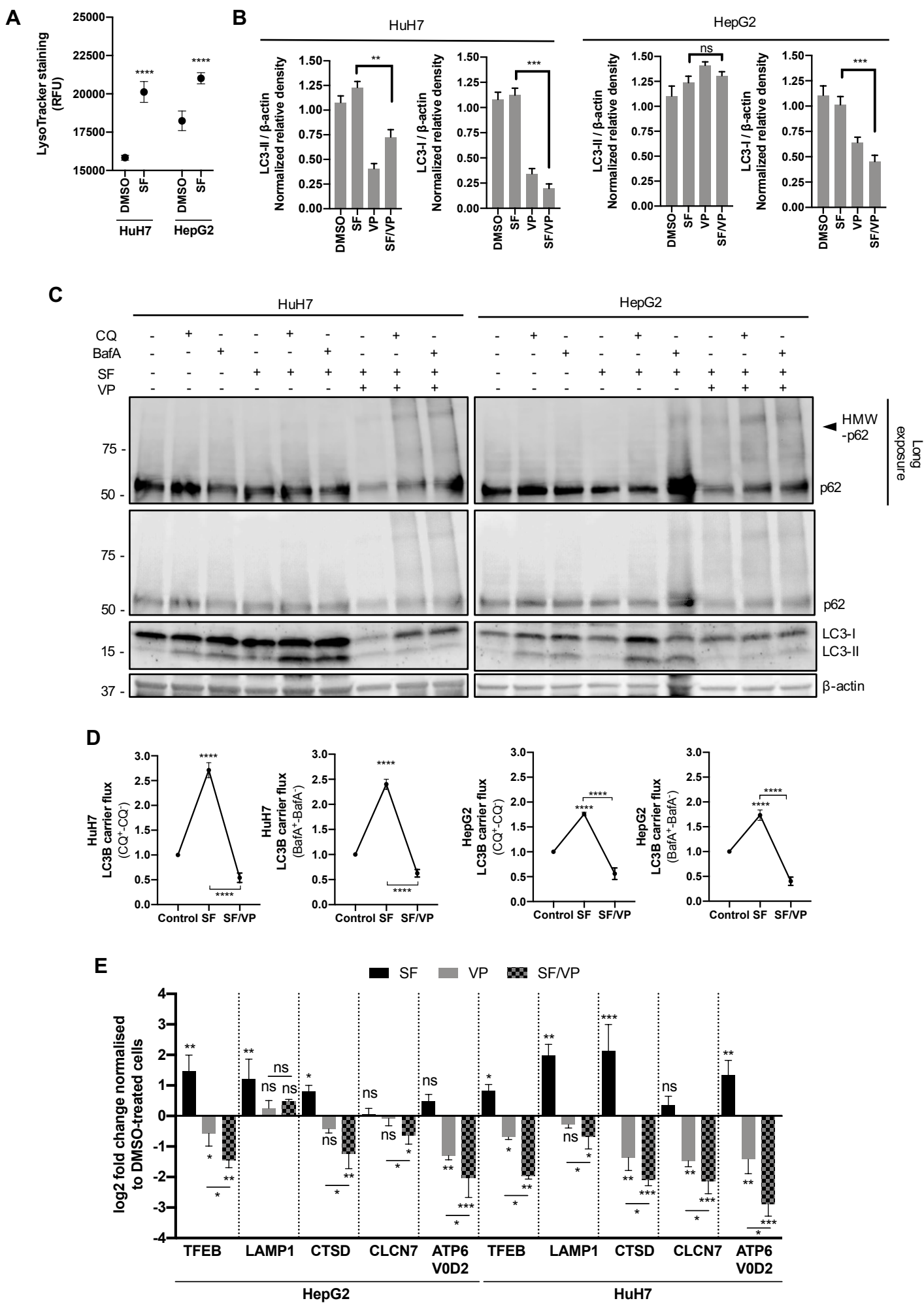

Supplement: Supplementary file 5 — Supplementary Figure 4 [file 41419_2019_1989_MOESM5_ESM.pdf]

**A**

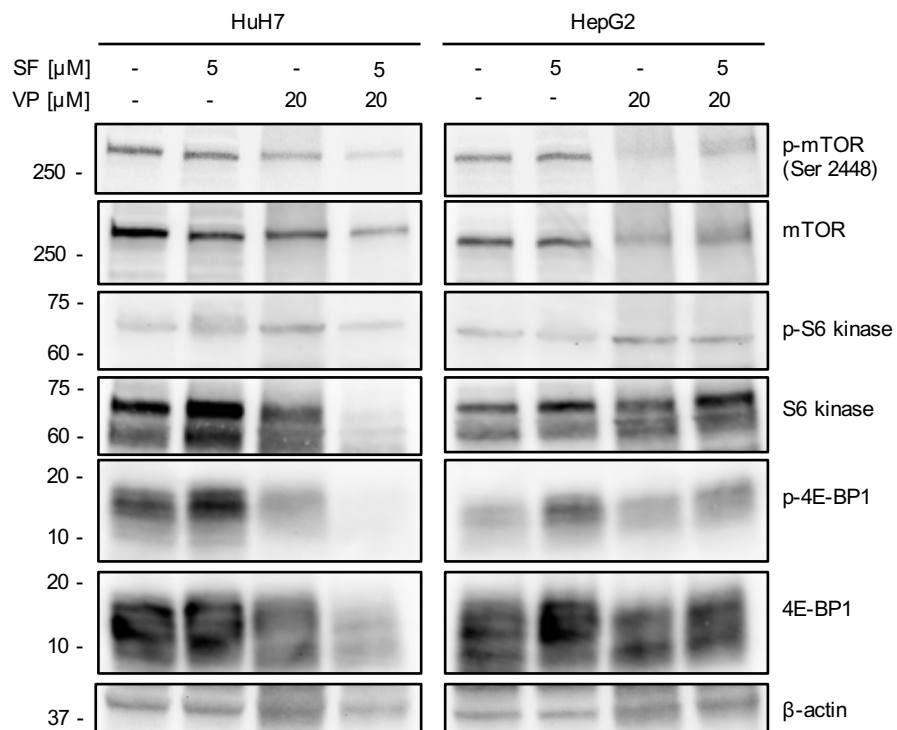

**B**

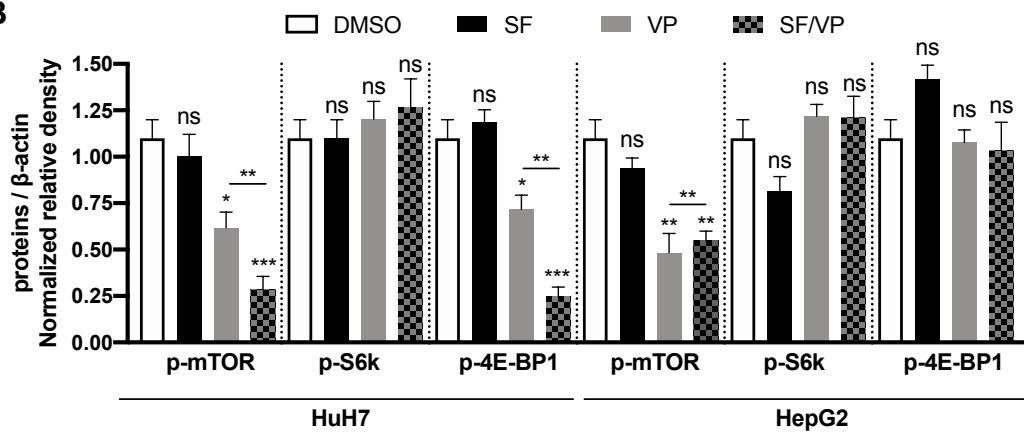

**C**

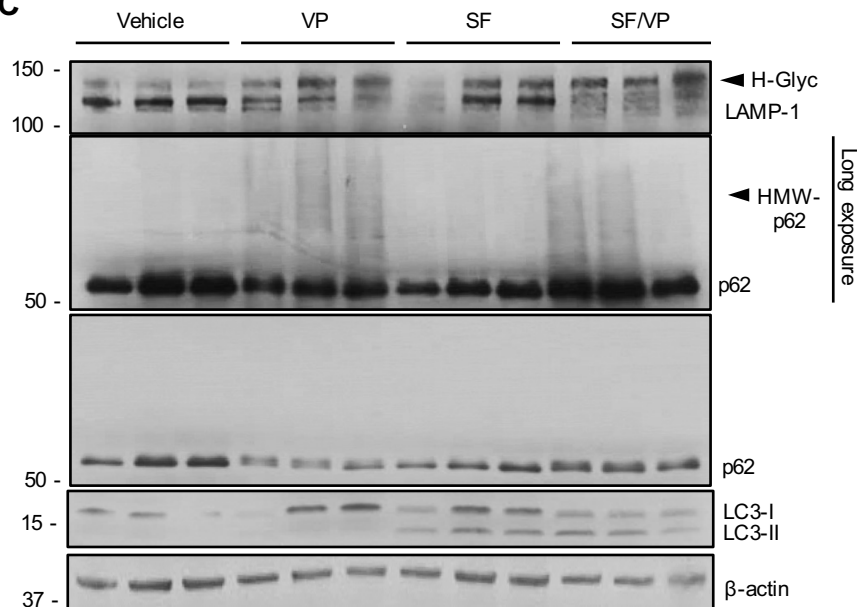

Supplement: Supplementary file 6 — Supplementary Figure 5 [file 41419_2019_1989_MOESM6_ESM.pdf]

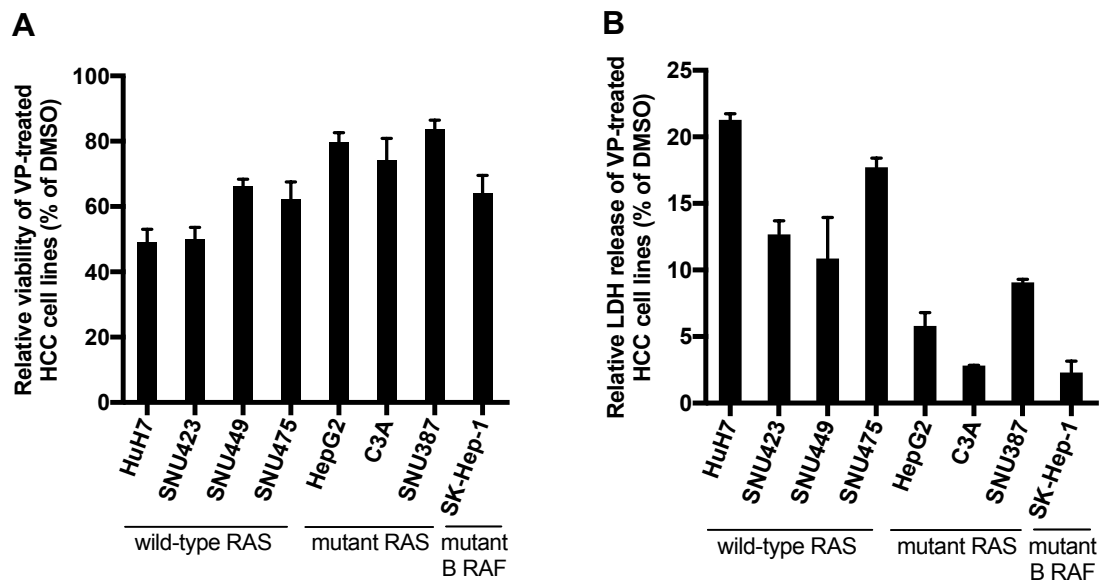

Supplement: Supplementary file 7 — Supplementary Figure 6 [file 41419_2019_1989_MOESM7_ESM.pdf]
